# AAV2/8-humanFOXP3 gene therapy shows robust anti-atherosclerosis efficacy in LDLR-KO mice on high cholesterol diet

**DOI:** 10.1186/s12967-015-0597-7

**Published:** 2015-07-18

**Authors:** M Cao, S A Theus, K D Straub, J A Figueroa, L Mirandola, M Chiriva-Internati, P L Hermonat

**Affiliations:** Central Arkansas Veterans Healthcare System, 111J, 4300 West 7th Street, Little Rock, AR 72205 USA; Division of Hematology and Oncology, Department of Internal Medicine, Texas Tech University Health Sciences Center, School of Medicine, Lubbock, TX 79415 USA; Kiromic LLC, Lubbock, TX USA

## Abstract

Inflammation is a key etiologic component in atherogenesis. Previously we demonstrated that adeno-associated virus (AAV) 2/8 gene delivery of Netrin1 inhibited atherosclerosis in the low density lipoprotein receptor knockout mice on high-cholesterol diet (LDLR-KO/HCD). One important finding from this study was that FOXP3 was strongly up-regulated in these Netrin1-treated animals, as FOXP3 is an anti-inflammatory gene, being the master transcription factor of regulatory T cells. These results suggested that the FOXP3 gene might potentially be used, itself, as an agent to limit atherosclerosis. To test this hypothesis AAV2/8 (AAV)/hFOXP3 or AAV/Neo (control) gene therapy virus were tail vein injected into the LDLR-KO/HCD animal model. It was found that hFOXP3 gene delivery was associated with significantly lower HCD-induced atherogenesis, as measured by larger aortic lumen cross sectional area, thinner aortic wall thickness, and lower aortic systolic blood velocity compared with Neo gene-HCD-treated controls. Moreover these measurements taken from the hFOXP3/HCD-treated animals very closely matched those measurements taken from the normal diet (ND) control animals. These data strongly suggest that AAV/hFOXP3 delivery gave a robust anti-atherosclerosis therapeutic effect and further suggest that FOXP3 be examined more stringently as a therapeutic gene for clinical use.

## Background

Inflammation is now known to be a key regulatory process that is common denominator among several risk factors for atherosclerosis, in addition to accompanying and associated altered arterial biology [1, 2]. Furthermore, it appears that both the innate and adaptive arms of the immune system may also be involved in this overall inflammatory trend which is implicated in atherosclerosis [3–7]. We have carried out various therapeutic adeno-associated virus (AAV)-based gene therapy studies in an animal model of atherosclerosis (low-density lipoprotein receptor-knockout mouse on high cholesterol diet, LDLR-KO HCD), towards the specific goal of regulating the arterial immune cell infiltrate status with immuno-suppressive cytokines and leukocyte chemo-attractant/repellant chemokine genes, and thereby inhibiting atherosclerosis [8–15].

We recently published a study that demonstrated that AAV/Netrin1 systemic gene delivery was able to inhibit atherosclerosis in LDLR-KO mice on HCD [13]. This was shown by high resolution ultrasound (HRUS) measurements of aortic lumen cross-sectional area, wall thickness, and systolic blood velocity. All of these measurements indicated that the Netrin1 gene delivery resulted in significantly lower atherosclerosis. However, upon analysis of the expression of various genes by Q-PCR we discovered that both Forkhead box P3’s (FOXP3) and CD25 expression were strongly up-regulated in the AAV/Netrin1-treated animals [13]. Of course both FOXP3 and CD25 are hallmark markers of regulatory T cells (Treg). However, the exact mechanism by which FOXP3 and CD25 are up-regulated by Netrin1 in aortas challenged with HCD remains to be determined.

Of these two genes, FOXP3, in particular, the master transcription factor of regulatory T cells (Treg), is intriguing as a therapeutic gene as the Treg phenotype is tied to FOXP3 expression, and Treg affect both innate and adaptive immunity [3–7]. It is the induction of the FOXP3 gene which results in giving an immune suppressive function to Treg precursor cells, and the removal of expression of this same gene in mature Treg cells results in loss of Treg lineage identity and a marked reduction in immunosuppressive properties [16–19]. Here we characterize the effect of AAV-based human (h)FOXP3 gene delivery, by systemic tail vein injection, to inhibit atherosclerosis in the LDLR-KO/HCD animal model. In this study we use the human (h)FOXP3 transgene rather than the mouse (m)Foxp3 version as the hFOXP3 and mFOXP3 proteins are 86% homologous, and the use of the human version brings us potentially one step closer to clinical trials.

## Methods

### Ethics statement

All experimental procedures were performed in accordance with protocols approved by the Institutional Animal Care and Usage Committee of the Central Arkansas Veterans Healthcare System, Research and Development, at Little Rock. The project was funded by a Veterans Administration Merit Review grant to PLH.

### AAV vector construction and virus generation

We directly addressed the hypothesis that hFOXP3 gene delivery can inhibit atherosclerosis by using AAV2/8 [AAV2 inverted terminal repeats (ITR) DNA combined with the AAV serotype 8 capsid] gene delivery. The human (h) FOXP3 cDNA was obtained from Open Biosystems and was ligated downstream from the cytomegalovirus immediate early promoter (CMVpr) within the gutted AAV vector dl3-97 to generate AAV/hFOXP3. The AAV/Neo vector has been described previously [8, 10–14]. AAV2/8 virus (AAV2 DNA in AAV8 virion) was produced using pDG8 helper and titered by dot blot analysis by standard methodologies [8, 10–14].

### Animal treatments

LDLR-KO mice (B6;129S7-*Ldlr*^*tm1Her*^/J) were purchased from Jackson Laboratories (Bar Harbor, ME, USA). Three groups of male mice, composed of ten animals each at 8 weeks old, were injected with AAV/Neo (positive control group), or AAV/hFOXP3 virus at a titer of 1 × 10^10^ e.g./ml via tail vein with 200 μL virus per mouse, two booster injections were followed at an interval of 5–6 days. High cholesterol diet (HCD) of 4% cholesterol and 10% cocoa butter diet (Harlan Teklad, Madison, Wis, USA) was provided from the first day of injection and maintained for the entire study period. Another group of mice fed with a ND was used as a negative control group. The normal background mouse chow was Harlan catalog #7012, and the HCD was #7012, 4% cholesterol/10% cocoa butter, custom formulated by Harlan. All experimental procedures conform to protocols approved by the Institutional Animal Care and Usage Committee of the Central Arkansas Veterans Health Care System at Little Rock.

### Ultrasound imaging

Ultrasound imaging was carried out using a Vevo 770 High-Resolution Imaging system (Visualsonics, Toronto, Canada) with a RMV 707B transducer having a center frequency of 30 MHz. Animal preparation was done as described earlier [20]. In brief, the mice were anesthetized using 1.5% isoflurane (Isothesia, Abbott Laboratories, Chicago, USA) with oxygen and laid supine out on a thermostatically heated platform. Abdominal hair was removed with a shaver and a chemical hair remover (Church & Dwight Co, Inc., NJ, USA). A pre-warmed transducing gel (Medline Industries, Inc., Mundelein, USA) was spread over the skin as a coupling medium for more accurate measurements. Two general levels of the vessel were visualized: thoracic region—below the aortic arches to the diaphragm and then the renal region—the upper abdominal region to the iliac bifurcation. Image acquisition was started on B-mode, where, a long axis view was used to visualize the length of the aorta. Then the scan head probe was turned 90° for a short-axis view to visualize the cross-sectional area of the aorta. Individual frames and cine loops (300 frames) were acquired at all levels of the aorta, and included both the long axis and short axis view and recorded at distances of 1 mm throughout the length of the aorta. Measurement of the flow velocity, orientation of the abdominal aorta by ultrasound, was accomplished by tilting the platform and the head of mouse down with the transducer probe towards the feet and tail of the mouse. This positioning resulted in the Doppler angle to be less than 60° for accurate measurements of blood flow velocity in the pulse-wave Doppler (PW) mode within abdominal aorta. Off-line measurements and data analysis was performed using the customized version of Vevo770 Analytical Software from both the longitudinal and transverse images. The complete imaging for each mouse lasted for about 25–30 min.

### Measurement of plasma cholesterol

Total plasma cholesterol of AAV/Neo and AAV/FOXP3 mice were measured by VetScan VS2 (Abaxis, Union City, CA, USA) at the Veterans Animal Laboratory (VAMU).

### Atherosclerotic lesion analysis by direct visualization

Whole dissected aortas were fixed in 10% buffered formalin, inspected under a dissecting microscope and any small pieces of adventitial fat that remained attached were removed very carefully without disturbing the aorta itself and the internal lipid accumulations/plaque. Unstained small animal aortas are normally translucent but show lipid deposition as white areas [21, 22]. Aortas were then photographed under natural light using a 10 megapixel digital camera (Nikon, Japan).

### Observation of atherosclerosis by histology

Twenty weeks after first injection of virus and on HCD, mice were killed by CO_2_ exposure. Entire aortas, including the aortic arches, thoracic and abdominal aortas, were removed. The aorta was flushed with saline solution and fixed in 10% neutral buffered formalin (Sigma). After 24 h, the fixed tissue was used for paraffin embedding and sectioning for histological analysis. Finally representative sections were hematoxylin and eosin-stained.

### Statistics

Parameters were analyzed with statistics software SPSS 16.0 by nonparametric ANOVA test. If differences were detected between means, Newman–Keuls test was used for multiple comparisons. Difference were considered as significant if *P* < 0.05.

## Results

### AAV vectors and animal treatments

**Figure 1:**
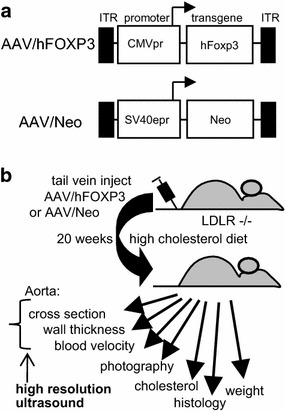
Vector structure and experimental overview. **a** The structure of the AAV virus vectors. **b** The overall structure of the study. The experimental details are provided in the “Methods” section.

**Figure 2:**
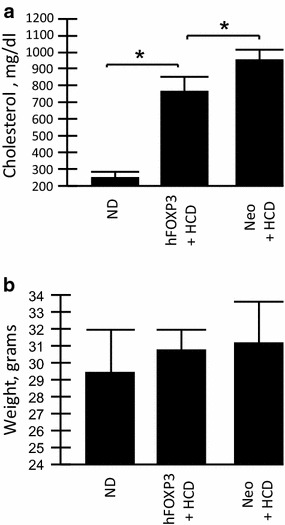
Animal characterization. **a** The levels of total cholesterol at week 20. **b** The animal weights at the end (week 20) of the experiment. The animals never received fasting conditions. *p* values of <0.05 refer to the student t test, are indicated by a “*asterisk*”, and also indicate statistical significance.

We directly addressed the hypothesis that hFOXP3 gene delivery can inhibit atherosclerosis by using AAV2/8 [AAV2 inverted terminal repeats (ITR) DNA combined with the AAV serotype 8 capsid] gene delivery. The AAV2/8-hFOXP3 was delivered by tail vein injection and the animals placed on HCD (4% cholesterol, 10% cocoa butter). Another animal group received AAV2/8-Neo virus delivered by tail vein injection and also placed on HCD, which served as positive controls. Lastly, a group of animals received only a ND and served as negative controls. The animals were then analyzed by high resolution ultrasound and harvested at 20 weeks post-injection/post HCD initiation. Figure 1a shows the general structure of the AAV vectors used Figure 1b shows the overall structure of the experiments. We have previously demonstrated specific delivery and expression of multiple transgenes into the aorta by AAV2/8 (AAV2 DNA inside the AAV8 capsid), utilizing the CMVpr transcriptional promoter, delivered by tail vein injection, to give expression levels 1.7-2.3% that of β-actin [10, 11]. Figure 2a shows that the blood cholesterol levels were high in both groups on HCD compared to the ND control. However, the AAV/hFOXP3-HCD and AAV/Neo-HCD treated animal were statistically different, with the AAV/hFOXP3-HCD group having a slightly lower cholesterol level. In Figure 2b, animal weights were statistically similar in all groups.

### Analysis of aortic structure

**Figure 3:**
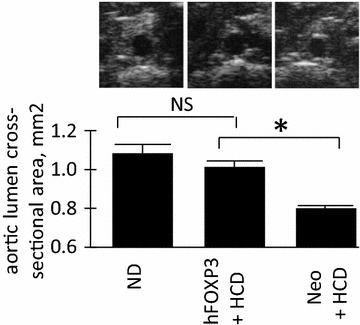
Aortic lumen cross-sectional area measurement. High resolution ultrasound (HRUS) was used to measure the cross-sectional area for the aortas in 6–8 animals from each animal group by HRUS with representative captured images from the analysis shown just above. Note that the AAV/hFOX3P-HCD animals had a larger cross sectional luminal area than the AAV/Neo-HCD animals. Also note that the AAV/hFOX3P-HCD and the ND groups were statistically the same. *P* values of <0.05 refer to the student t test, are indicated by a “*asterisk*”, and also indicate statistical significance.

**Figure 4:**
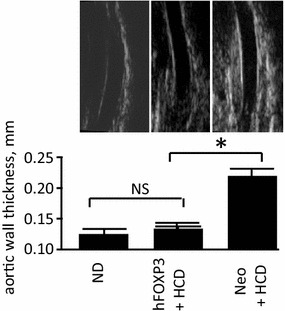
Aortic wall thickness measurement. HRUS was used to measure the wall thickness of the aorta. Shown is a quantification of the wall thickness of the aorta (thoracic region) of indicated groups with representative captured images from the analysis shown just above. Note that the AAV/hFOXP3-HCD animals have a significantly thinner wall thickness than the AAV/Neo-HCD animals. Also note that the AAV/hFOX3P-HCD and the ND groups were statistically the same. *p* values of < 0.05 refer to the student t test, are indicated by a “*asterisk*”, and also indicate statistical significance.

**Figure 5:**
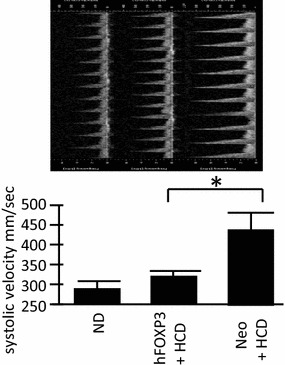
Systolic blood velocity measurement. High resolution ultrasound (HRUS) was used to quantify blood flow velocities in the luminal center of the aorta in 6–8 animals from each group with representative captured images from the analysis shown just above. Note that the AAV/hFOXP3-HCD animals have a much lower blood velocity than the AAV/Neo-HCD animals. Also note that the AAV/hFOX3P-HCD and the ND groups were very similar in peak systolic blood velocity. *p* values of < 0.05 refer to the student t test, are indicated by a “*asterisk*”, and also indicate statistical significance.

High resolution ultrasound (HRUS) was then used to analyze the aortas of at least eight animals per group. Figure 3 shows that the cross-sectional area of the lumens of the aortas was significantly larger (*p* < 0.05) in the hFOXP3/HCD-treated animals than the Neo/HCD-treated animals by HRUS. Moreover, the lumens of the aortas in the hFOXP3/HCD-treated animals versus the ND control animals (negative control) were statistically the same (not significant, NS). This evidence of robust efficacy was surprising to us as Netrin1 gene delivery, while showing efficacy, did not give this higher level of efficacy. Moreover, further HRUS analysis, as shown in Figure 4, indicated that aortic wall thickness was significantly thinner (*p* < 0.05) in the hFOXP3/HCD-treated animals than the Neo/HCD-treated, positive control animals. Additionally, the wall thickness of the aorta in the hFOXP3/HCD-treated animals and the ND control animals were statistically the same (not significant, NS). Thus, this additional evidence of robust efficacy fully supports the lumen area analysis. Figure 5 shows that the systolic blood velocity (systolic pulse wave velocity) in the aorta of the hFOXP3/HCD-treated animals was significantly lower (*p* < 0.05 than the Neo/HCD-treated animals, again consistent with a lower level of atherosclerosis. In this additional measurement the wall thickness of the aorta in the hFOXP3/HCD-treated animals and the ND control animals were statistically significant. However, it can also be easily observed that the systolic blood velocity of the hFOXP3/HCD-treated animals was much closer (lower) to the ND animals than to the Neo/HCD-treated animals. The correlation between low systolic blood velocity with thinner aortic wall thickness and larger aortic lumens we observed here matches the correlations we have seen in our other anti-atherosclerosis gene delivery studies in our studies of LDLR-KO mice on 4% cholesterol, 10% cocoa butter HCD [8, 10–14]. This again indicated that the hFOXP3 gene delivery did provide high level of efficacy against HCD-associated atherosclerosis.

### Visual inspection of aortas

**Figure 6:**
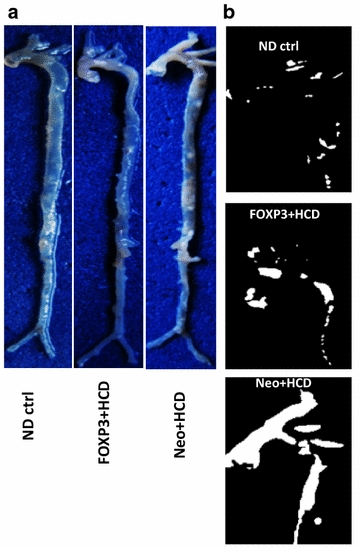
Visual inspection of representative aortas. **a** Aortas from the indicated animals were buffered formalin-fixed, cleaned and photographed. Note that the AAV/Neo-treated HCD aorta displays much higher amounts of lipid accumulation (*white areas*) than ether the AAV/hFOXP3-HCD-treated animals. **b** Shown is a PowerPoint black-white 25% enhancement of the aortic arch of the three aortas. Note that the AAV/Neo-treated HCD aorta displays a much more extensive white area than ether the AAV/hFOXP3-HCD-treated or ND animals.

We further studied the effects of the transgene hFOXP3 transgene on the aorta challenged with HCD by visual inspection. It has already been documented that unstained small animal aortas show lipid deposition as white areas [21, 22]. Figure 6a shows representative unstained aortas from the indicated animal groups. It is readily visible that the AAV/Neo-HCD-treated animals have much more numerous areas of white in both the aortic arch and in the descending aorta than either the ND or AAV/hFOXP3-HCD-treated animals. As the aortic arch is particularly susceptible to atherosclerosis the aortic arches in these same aortas were further image enhanced using MS PowerPoint black-white 25% display as shown in Figure 6b. Again, consistent with the HRUS measurements, the AAV/hFOXP3-HCD-treated animal showed much less atherosclerosis than the AAV/Neo-HCD-treated animal, and was very similar to the ND animal.

### Histologic views of representative aortas

**Figure 7:**
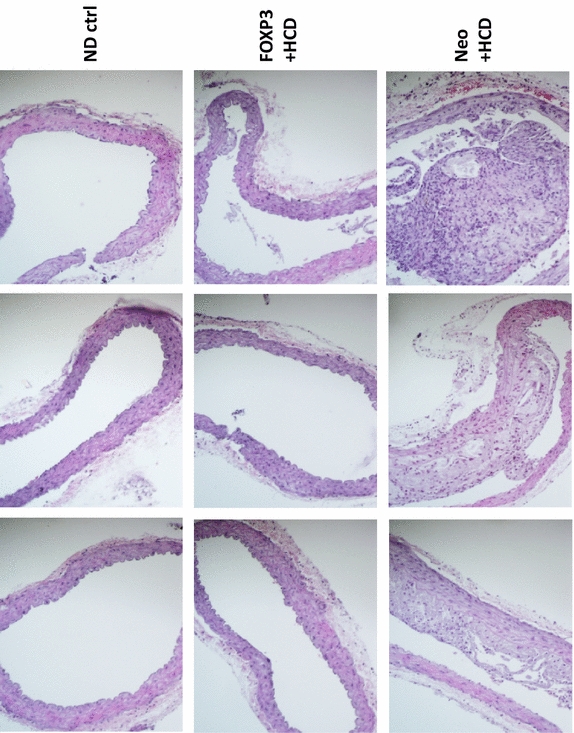
Histologic views of representative aortas. Aortas from each of three representative animals from the three indicated animal groups (indicated column) were buffered formalin-fixed, paraffin-embedded, sectioned and hematoxylin and eosin stained. Note that the higher level of atherosclerotic plaque in the AAV/Neo-HCD-treated group is readily apparent.

Finally, histologic sections were taken across the axis of the aortas to give representative cross sectional views. In Figure 7, representative hematoxylin and eosin-stained sections are shown, one each from each of three animals, from each of the three animal treatment groups. Note that the AAV/Neo-HCD group showed much higher levels of atherosclerotic plaque than either the ND or AAV/FOXP3-HCD group, consistent with the HRUS (Figures 3, 4, 5) and the direct visualization (Figure 6) analysis. The photomicrographs were further analyzed for smooth muscle layer thickness, but no statistical significance was seen. However, within the AAV/Neo-HCD animal group an increase in smooth muscle layer thickness of 61% (*p* < 0.05) was observed in regions associated with plaque compared to the opposing non-atherosclerotic region of the same micrograph.

## Discussion

Our earlier Netrin1 gene therapy study demonstrated that FOXP3 and CD25 were strongly overexpressed in aortas compared to controls [13], and that this overexpression was associated with significantly lower atherosclerosis. From these results we developed the hypothesis that the Treg phenotype, specifically induced by AAV/FOXP3 gene delivery, would show an inhibition of atherogenesis in our LDLR-KO/HCD model. This study has demonstrated that our hypothesis was correct and that CMVpr-FOXP3-gene delivery by AAV2/8 vector results in a robust protection of aortas from developing atherosclerosis in the LDLR-KO/HCD model. The protection afforded by the FOXP3-treated animals on HCD was shown by the finding that multiple aortic parameters were *not statisticallydifferent* from the ND control group. These data suggest that the FOXP3 gene gives a high level of efficacy against HCD-induced atherosclerosis in the well-established LDLR/KO-HCD animal model. None of our other therapeutic gene therapy in LDLR-KO/HCD mouse studies has given multiple aortic parameters which were statistically the same as the ND control group [8, 10–14]. This includes our “gold standard” IL10 gene, which has been utilized by at least three groups for inhibiting atherosclerosis [12, 15, 23–27].

Very likely the efficacious ability of FOXP3 lies in its role as the master transcription factor for the Treg development and phenotype. It is known that the loss of FOXP3 expression (or mutation FOXP3) results in increases in chronic autoimmunity [18]. The phenotype of FOXP3 knockouts gives the “scurfy” phenotype in mice and in humans generates the X-linked autoimmunity–allergic dysregulation and immuno-dysregulation, X-linked syndromes [28, 29]. Tregs are also critical for lowering excess inflammation and giving tolerance to gut commensal microbes [30]. However, if present in extreme excess, Tregs may allow for dysplastic and malignant cell growth and chronic infections through the governance of limited anti-tumor surveillance or limited anti-pathogenic organism immune responses [30–32].

As we age it is clear that inflammation increases and becomes a major threat to health [33–35]. Thus, the use of Tregs, the promotion of the Treg phenotype, is one possible approach to broadly limit this increasing inflammation, in the aged population. The use of AAV-based FOXP3 gene therapy, to promote the overall Treg phenotype, could be an effective therapeutic gene and agent against inflammation and diseases of the elderly. It is very important that the forced induction of the FOXP3 gene results in giving an immune suppressive function to Treg precursor cells, and that the removal of expression of this same gene in mature Treg cells results in loss of Treg lineage identity and a marked reduction in immunosuppressive properties [16, 19].

One possible mechanism for the AAV/hFOXP3-HCD animal group cholesterol levels being lower than the AAV/Neo-HCD group is the likelihood that Foxp3 is known to induce both transforming growth factor beta 1 (TGFβ1) and interleukin 10 (IL10). Both of these are known to be associated with lower cholesterol levels and both of TGFβ1 and IL10 are known to inhibit the development of atherosclerosis. Both inhibit macrophage trafficking into the arterial wall intima and thereby inhibit macrophage accumulation and foam cell formation. Additionally it has been reported by Klingenberg et al that Foxp3 expression is linked with lower cholesterol levels, as we see here [36]. Thus, in summary, our data on FOXP3 gene delivery, and, in addition, the general literature on FOXP3 suggests that it will likely be a robust therapeutic gene for the treatment of atherosclerosis
